# Investigating the Association between Alcohol and Risk of Head and Neck Cancer in Taiwan

**DOI:** 10.1038/s41598-017-08802-4

**Published:** 2017-08-29

**Authors:** Cheng-Chih Huang, Jenn-Ren Hsiao, Wei-Ting Lee, Yao-Chou Lee, Chun-Yen Ou, Chan-Chi Chang, Yu-Cheng Lu, Jehn-Shyun Huang, Tung-Yiu Wong, Ken-Chung Chen, Sen-Tien Tsai, Sheen-Yie Fang, Jiunn-Liang Wu, Yuan-Hua Wu, Wei-Ting Hsueh, Chia-Jui Yen, Shang-Yin Wu, Jang-Yang Chang, Chen-Lin Lin, Yi-Hui Wang, Ya-Ling Weng, Han-Chien Yang, Yu-Shan Chen, Jeffrey S. Chang

**Affiliations:** 1 Department of Otolaryngology, National Cheng Kung University Hospital, College of Medicine, National Cheng Kung University, 138 Sheng Li Road, Tainan, 70456 Taiwan; 2 Division of Plastic and Reconstructive Surgery, Department of Surgery, National Cheng Kung University Hospital, College of Medicine, National Cheng Kung University, 138 Sheng Li Road, Tainan, 70456 Taiwan; 3 Department of Stomatology, National Cheng Kung University Hospital, College of Medicine, National Cheng Kung University, 138 Sheng Li Road, Tainan, 70456 Taiwan; 4 Department of Radiation Oncology, National Cheng Kung University Hospital, College of Medicine, National Cheng Kung University, 138 Sheng Li Road, Tainan, 70456 Taiwan; 5 Division of Hematology/Oncology, Department of Internal Medicine, National Cheng Kung University Hospital, College of Medicine, National Cheng Kung University, 138 Sheng Li Road, Tainan, 70456 Taiwan; 6 National Institute of Cancer Research, National Health Research Institutes, 1F No 367, Sheng-Li Road, Tainan, 70456 Taiwan; 7 Department of Nursing, National Cheng Kung University Hospital, College of Medicine, National Cheng Kung University, 138 Sheng Li Road, Tainan, 70456 Taiwan

## Abstract

Although alcohol is an established risk factor of head and neck cancer (HNC), insufficiencies exist in the literature in several aspects. We analyzed detailed alcohol consumption data (amount and type of alcoholic beverage) of 811 HNC patients and 940 controls to evaluate the association between alcohol and HNC by HNC sites and by genotypes of *ADH1B* and *ALDH2*. Alcohol was associated with an increased HNC risk in a dose-response relationship, with the highest risk observed for hypopharyngeal cancer, followed by oropharyngeal and laryngeal cancers. Liquor showed a stronger positive association with HNC than beer and wine. The highest HNC risk occurred in individuals with the slow *ADH1B* and slow/non-functional *ALDH2* genotype combination. In our study population, 21.8% of HNCs, 55.7% of oropharyngeal cancers, and 89.1% of hypopharyngeal cancers could be attributed to alcohol. Alcohol accounted for 47.3% of HNCs among individuals with the slow *ADH1B* and slow/non-functional *ALDH2* genotype combination. The HNC risk associated with alcohol became comparable to that of never/occasional drinkers after ten or more years of cessation from regular alcohol drinking. In conclusion, alcohol use is associated with an increased HNC risk, particularly for individuals with slow ethanol metabolism. HNC incidence may be reduced by alcohol cessation.

## Introduction

Head and neck cancer (HNC), including cancers of the oral cavity, oropharynx, hypopharynx, and larynx, is the fifth most common cancer in the world^1^. Besides cigarette, betel quid, and human papillomavirus (specifically for oropharyngeal cancer), alcohol is an established risk factor of HNC^2, 3^; however, detailed assessment is needed to characterize the association between alcohol and HNC by dose-response, by different anatomic sites, by alcoholic beverage type, and by ethanol metabolizing gene polymorphisms, and to quantify the impact of alcohol cessation.

Although several aspects of alcohol consumption have been investigated in HNC studies, uncertainties still exist. A meta-analysis reported that compared to oral cancer and laryngeal cancer, pharyngeal cancer showed a stronger association with alcohol^4^. Studies included in this meta-analysis analyzed oropharyngeal and hypopharyngeal cancers together as pharyngeal cancer. Because oropharyngeal cancer but not hypopharyngeal cancer is strongly associated with HPV, combining oropharyngeal cancer and hypopharyngeal cancer may not be ideal for investigating the impact of alcohol by HNC sites. Many studies have investigated the association between alcoholic beverage type (beer, wine, and liquor) and HNC with inconsistent results^5–13^. A meta-analysis reported the dose-response relationship between alcohol consumption and various cancers, including HNC^14^. However, most studies of HNC included in that meta-analysis were from Western countries and only a few studies were conducted in East Asian populations. East Asian countries, including Taiwan, have a high prevalence of individuals with slow ethanol metabolism^15^. Individuals with slow ethanol metabolism may have a dose-response relationship between alcohol and HNC risk that is different from that in individuals with normal ethanol metabolism. Alcohol cessation is known to reduce HNC risk, although it is unclear whether this risk reduction may differ by alcohol consumption level before alcohol cessation^16, 17^.

Acetaldehyde, a carcinogen, is produced from ethanol by alcohol dehydrogenase (ADH). Acetaldehyde is metabolized by aldehyde dehydrogenase (ALDH) to non-toxic acetate^18^. Ethanol metabolizing gene polymorphisms may determine the speed of ethanol metabolism and modulate an individual’s susceptibility to the carcinogenic effect of alcohol. ADH1B, encoded by the gene *ADH1B*, is a major enzyme that converts ethanol to acetaldehyde. *ADH1B* has three alleles determined by codons 48 (Arg48His; rs1229984) and 370 (Arg370Cys; rs2066702): the ADH1B*1 allele = arginine at codon 48 (Arg48) + arginine at codon 370 (Arg370), the ADH1B*2 allele = histidine at codon 48 (His48) + Arg370, and the ADH1B*3 allele = Arg48 + cysteine at codon 370 (Cys370)^19^. The ADH1B*2 allele is prevalent among Pacific Rim Asians (60–85%)^20^ and the ADH1B*2/*2 genotype encodes an enzyme that converts ethanol to acetaldehyde 40 times faster than the enzyme encoded by the ADH1B*1/*1 genotype^21^. The ADH1B*3 allele occurs mostly among individuals of African descent and generates an enzyme with 15% higher activity than the one produced by the *1 allele^22^. The *ALDH2* gene encodes the enzyme ALDH2, which converts acetaldehyde to acetate. Rs671 is a well-known functional single nucleotide polymorphism (SNP) of *ALDH2* with two alleles: *1 = glutamate at codon 504 and *2 = lysine at codon 504^23^. ALDH2*2/*2 genotype encodes an enzyme with 4% activity compared to the enzyme produced by the ALDH2*1/*1 wild type genotype, while the heterozygous ALDH2*1/*2 genotype encodes an enzyme with <50% enzyme activity^24^. The ALDH2*2 allele is uncommon (prevalence < 5%) among the Europeans and Africans^25^, while 30–50% of the East Asians (Chinese, Japanese, Korean, and Taiwanese) carry at least one copy of the *2 allele^26^. Although the association between ethanol metabolizing genes and HNC has been investigated, only a few studies have examined the combined effect of *ADH1B* and *ALDH2* ^27, 28^.

We previously published an article on the interplay between genetic polymorphisms of *ADH1B* and *ALDH2*, alcohol drinking, and poor oral hygiene in the development of HNC using data from an ongoing HNC case-control study^28^. Since then we nearly doubled our sample size for the current analysis, which aimed to fill the above-mentioned insufficiencies in the literature on the association between alcohol and HNC.

## Results

This study included 811 HNC patients and 940 controls for the analysis with alcohol consumption status (never, former regular, current regular) and frequency. Because information on alcoholic beverage type was not collected before March 20, 2011, the analyses with intensity (never, light, moderate, and heavy) and alcoholic beverage type included fewer subjects (737 HNC cases and 872 controls). Supplementary Table 1 shows the distribution of the clinical diagnoses among controls. Cases and controls were similar in the distributions of age (*p* = 0.12) and sex (*p* = 0.45) (Table 1). Controls were more highly educated and consumed more vegetables and fruits than cases (*p* < 0.0001). Cases consumed more betel quid and cigarette than controls (*p* < 0.0001).

**Table 1:** Demographic and lifestyle characteristics of the head and neck cancer patients and control subjects

Characteristics	Case N = 811 n (%)	Control N = 940 n (%)	*P*
Age (years)			
Mean (SE)	55.4 (0.4)	54.6 (0.3)	0.12
Sex			
Men	761 (93.8)	890 (94.7)	0.45
Women	50 (6.2)	50 (5.3)	
Education			
≦Elementary school	224 (27.6)	163 (17.3)	<0.0001
Junior high	240 (29.6)	166 (17.7)	
High school/Technical school	267 (32.9)	336 (35.7)	
Some college or more	80 (9.9)	275 (29.3)	
Betel quid chewing			
Never	233 (28.7)	676 (71.9)	<0.0001
Former	310 (38.2)	171 (18.2)	
Current	268 (33.1)	92 (9.8)	
Unknown	0 (0.0)	1 (0.1)	
Never	233 (28.7)	676 (71.9)	0.0001
0.1–9.9 pack-years	119 (14.7)	93 (9.9)	
10.0–19.9 pack-years	102 (12.6)	49 (5.2)	
20.0–29.9 pack-years	90 (11.1)	39 (4.2)	
30.0 or more pack-years	247 (30.4)	80 (8.5)	
Unknown	20 (2.5)	3 (0.3)	
Pack-years (SE)	26.3 (1.3)	7.5 (0.7)	<0.0001
Cigarette smoking			
Never	111 (13.7)	313 (33.3)	<0.0001
Former	153 (18.9)	189 (20.1)	
Current	546 (67.3)	437 (46.5)	
Unknown	1 (0.1)	1 (0.1)	
Never	313 (33.3)	111 (13.7)	<0.0001
0.1–9.9 pack-years	38 (4.7)	76 (8.1)	
10.0–19.9 pack-years	80 (9.8)	121 (12.9)	
20.0–29.9 pack-years	137 (16.9)	119 (12.7)	
30.0 or more pack-years	434 (53.5)	305 (32.4)	
Unknown	11 (1.4)	6 (0.6)	
Pack-years (SE)	35.6 (1.0)	22.8 (0.9)	<0.0001
Vegetable intake			
Non-daily	149 (18.4)	82 (8.7)	<0.0001
Daily	660 (81.4)	858 (91.3)	
Unknown	2 (0.2)	0	
Fruit intake			
Non-daily	565 (69.7)	452 (48.1)	<0.0001
Daily	243 (29.9)	487 (51.8)	
Unknown	3 (0.4)	1 (0.1)	

Abbreviations: N = number; SE = standard error.

Ever regular alcohol drinking was associated with an increased HNC risk (OR = 1.62, 95% CI: 1.28–2.06), particularly for current drinker (OR = 1.81, 95% CI: 1.41–2.34) and daily drinker (OR = 1.72, 95% CI: 1.32–2.23) (Table 2). Alcohol showed a positive dose-response relationship with HNC risk, with moderate (OR = 1.47, 95% CI: 1.02–2.11) and heavy drinking (OR = 2.21, 95% CI: 1.61–3.02) significantly associated with an increased HNC risk. In addition, every 10 grams/day of alcohol consumption was associated with a 4% increase in HNC risk (OR = 1.04, 95% CI: 1.02–1.05). Alcohol consumption showed the strongest positive association with hypopharyngeal cancer, followed by cancers of oropharynx, larynx, and oral cavity. For hypopharyngeal cancer, an increased risk was observed even for low frequency or low amount of drinking (weekly drinking: OR = 13.47, 95% CI: 3.98–45.63; light drinking: OR = 6.63, 95% CI: 2.03–21.63). For oropharyngeal cancer, an increased risk could be observed for weekly drinking (OR = 2.92, 95% CI: 1.35–6.34) or moderate drinking (OR = 4.04, 95% CI: 1.86–8.81). For laryngeal cancer, an increased risk was only observed for daily drinking (OR = 1.91, 95% CI: 1.10–3.30) or heavy drinking (OR = 3.44, 95% CI: 1.85–6.41). For oral cancer, a non-statistically increased risk was observed for current drinker (OR = 1.29, 95% CI: 0.97–1.73) as the 95% CI included 1.

**Table 2:** The association between alcohol drinking and risk of head and neck cancer overall and by sites.

Alcohol drinking^a^	Controls n (%)	Head and neck cancer n (%)	OR (95% CI)^b^	Oral cavity n (%)	OR (95% CI)^b^	Oro- pharynx n (%)	OR (95% CI)^b^	Hypo- pharynx n (%)	OR (95% CI)^b^	Larynx n (%)	OR (95% CI)^b^
Never + occasional	517 (55.0)	263 (32.4)	Referent	195 (38.3)	Referent	29 (24.6)	Referent	4 (4.5)	Referent	35 (36.8)	Referent
Former regular	109 (11.6)	111 (13.7)	1.14 (0.80–1.62)	61 (12.0)	0.77 (0.51–1.17)	20 (16.9)	2.83 (1.39–5.76)	19 (21.3)	14.02 (4.38–44.85)	11 (11.6)	0.86 (0.40–1.85)
Current regular	314 (33.4)	437 (53.9)	1.81 (1.41–2.34)	253 (49.7)	1.29 (0.97–1.73)	69 (58.5)	4.23 (2.38–7.52)	66 (74.2)	21.55 (7.36–63.15)	49 (51.6)	1.84 (1.09–3.11)
Ever regular (Former regular + current regular)	423 (45.0)	548 (67.6)	1.62 (1.28–2.06)	314 (61.7)	1.14 (0.87–1.50)	89 (75.4)	3.80 (2.19–6.60)	85 (95.5)	19.17 (6.64–55.39)	60 (63.2)	1.51 (0.92–2.48)
**Frequency**											
Never	454 (48.3)	238 (29.3)	Referent	178 (35.0)	Referent	26 (22.0)	Referent	4 (4.5)	Referent	30 (31.6)	Referent
Monthly	63 (6.7)	25 (3.1)	1.05 (0.61–1.81)	17 (3.3)	0.92 (0.49–1.73)	3 (2.6)	1.31 (0.36–4.73)	0	—	5 (5.2)	1.81 (0.64–5.15)
Weekly	126 (13.4)	83 (10.2)	1.32 (0.92–1.90)	49 (9.6)	0.94 (0.61–1.46)	13 (11.0)	2.92 (1.35–6.34)	12 (13.5)	13.47 (3.98–45.63)	9 (9.5)	1.05 (0.46–2.40)
Daily	291 (31.0)	447 (55.1)	1.72 (1.32–2.23)	250 (49.1)	1.15 (0.85–1.55)	74 (62.7)	4.38 (2.39–8.06)	73 (82.0)	18.83 (6.45–54.99)	50 (52.6)	1.91 (1.10–3.30)
Unknown	6 (0.6)	18 (2.2)	—	15 (3.0)	—	2 (1.7)	—	0 (0.0)	—	1 (1.1)	—
**Amount**											
Never	435 (49.9)	222 (30.1)	Referent	165 (36.0)	Referent	24 (21.4)	Referent	4 (5.0)	Referent	29 (33.0)	Referent
Light	192 (22.0)	105 (14.3)	1.08 (0.77–1.50)	68 (14.9)	0.85 (0.58–1.25)	13 (11.6)	1.90 (0.86–4.19)	13 (16.5)	6.63 (2.03–21.63)	11 (12.5)	0.77 (0.36–1.66)
Moderate	103 (11.8)	109 (14.8)	1.47 (1.02–2.11)	66 (14.4)	1.13 (0.74–1.72)	17 (15.2)	4.04 (1.86–8.81)	19 (24.1)	14.54 (4.55–46.51)	7 (7.9)	0.86 (0.35–2.15)
Heavy	132 (15.1)	283 (38.4)	2.21 (1.61–3.02)	144 (31.4)	1.25 (0.87–1.80)	56 (50.0)	7.86 (3.95–15.63)	43 (54.4)	20.36 (6.75–61.36)	40 (45.5)	3.44 (1.85–6.41)
Unknown	10 (1.2)	18 (2.4)	—	15 (3.3)	—	2 (1.8)	—	0 (0.0)	—	1 (1.1)	—
Every 10 grams/day of ethanol			1.04 (1.02–1.05)		1.01 (1.00–1.03)		1.06 (1.03–1.09)		1.07 (1.03–1.10)		1.05 (1.02–1.08)

Abbreviations: N = number; CI = confidence interval; OR = odds ratio.

^a^Light: <14 grams/day; moderate: 14–42 grams/day; heavy: >42 grams/day.

^b^OR and 95% CI were calculated using unconditional logistic regression, adjusted for, age, sex, education, cigarette smoking (pack-year categories), and betel quid chewing (pack-year categories).

With the same amount of pure alcohol, liquor showed a stronger positive association with HNC risk (Table 3). For beer, an increased HNC risk was observed only for heavy drinking (OR = 2.56, 95% CI: 1.75–3.74). No significant association was observed between wine and HNC; however, the percentage of wine drinker was low. For liquor, an increased HNC risk could be seen at moderate drinking level (OR = 1.89, 95% CI: 1.25–2.85). For oropharyngeal and hypopharyngeal cancers, an increased risk could be seen with light drinking regardless of alcoholic beverage type, although stronger in magnitude for liquor than beer.

**Table 3:** The association between alcohol and head and neck cancer by alcohol type.

Type of alcohol^a^	Controls n (%)	All head and neck cancer n (%)	OR (95% CI)^b^	Oral cavity n (%)	OR (95% CI)^b^	Oro- pharynx n (%)	OR (95% CI)^b^	Hypo- pharynx n (%)	OR (95% CI)^b^	Larynx n (%)	OR (95% CI)^b^
**Beer**											
Never	435 (49.9)	222 (30.1)	Referent	165 (36.0)	Referent	24 (21.4)	Referent	4 (5.0)	Referent	29 (33.0)	Referent
Light	189 (21.7)	134 (18.2)	1.24 (0.90–1.71)	89 (19.4)	1.04 (0.72–1.51)	14 (12.5)	1.95 (0.89–4.30)	17 (21.5)	8.09 (2.55–25.59)	14 (15.9)	0.97 (0.47–2.00)
Moderate	87 (10.0)	100 (13.6)	1.29 (0.88–1.89)	54 (11.8)	0.80 (0.51–1.24)	21 (18.7)	4.61 (2.14–9.91)	12 (15.2)	9.39 (2.78–31.73)	13 (14.8)	1.71 (0.77–3.78)
Heavy	66 (7.6)	158 (21.4)	2.56 (1.75–3.74)	83 (18.1)	1.51 (0.98–2.34)	34 (30.4)	9.41 (4.51–19.67)	23 (29.1)	23.01 (7.16–73.93)	18 (20.4)	3.11 (1.49–6.48)
Did not drink beer but drank other types of alcohol	85 (9.8)	106 (14.4)	1.67 (1.14–2.44)	53 (11.6)	1.05 (0.67–1.67)	17 (15.2)	4.44 (2.04–9.67)	23 (29.1)	21.10 (6.65–66.96)	13 (14.8)	1.70 (0.79–3.68)
Unknown	10 (1.1)	17 (2.3)	—	14 (3.1)	—	2 (1.8)	—	0 (0.0)	—	1 (1.1)	—
Every 10 grams/day of ethanolfrom beer			1.01 (0.98–1.05)		1.00 (0.97–1.03)		1.03 (0.98–1.07)		1.05 (1.00–1.09)		1.01 (0.96–1.07)
Every 10 grams/day of ethanolfrom other types of alcoholic beverage			1.06 (1.02–1.10)		1.04 (1.00–1.08)		1.09 (1.04–1.15)		1.10 (1.03–1.17)		1.09 (1.03–1.15)
**Wine**											
Never	435 (49.9)	222 (30.1)	Referent	165 (36.0)	Referent	24 (21.4)	Referent	4 (5.0)	Referent	29 (33.0)	Referent
Light	47 (5.4)	28 (3.8)	1.28 (0.73–2.25)	16 (3.5)	0.83 (0.42–1.64)	8 (7.1)	5.86 (2.24–15.34)	2 (2.5)	6.84 (1.11–42.04)	2 (2.3)	0.98 (0.21–4.51)
Moderate	5 (0.6)	0 (0.0)	—	0 (0.0)	—	0 (0.0)	—	0 (0.0)	—	0 (0.0)	—
Heavy	1 (0.1)	5 (0.7)	4.11 (0.40–42.54)	3 (0.7)	4.10 (0.27–62.11)	0 (0.0)	—	1 (1.3)	53.25 (1.47- > 999.99)	1 (1.1)	8.60 (0.50–148.83)
Did not drink wine but drank other types of alcohol	375 (43.0)	465 (63.1)	1.58 (1.22–2.05)	260 (56.8)	1.09 (0.81–1.47)	78 (69.7)	4.04 (2.17–7.51)	72 (91.2)	13.75 (4.76–39.67)	55 (62.5)	1.61 (0.94–2.77)
Unknown	9 (1.0)	17 (2.3)	—	14 (3.1)	—	2 (1.8)	—	0 (0.0)	—	1 (1.1)	—
Every 10 grams/day of ethanol from wine			0.97 (0.85–1.11)		1.01 (0.87–1.18)		0.63 (0.15–2.60)		0.97 (0.73–1.27)		1.02 (0.85–1.22)
Every 10 grams/day of ethanol from other types of alcoholic beverage			1.04 (1.02–1.06)		1.01 (0.99–1.04)		1.06 (1.03–1.10)		1.07 (1.04–1.11)		1.05 (1.02–1.08)
**Liquor**											
Never	435 (49.9)	222 (30.1)	Referent	165 (36.0)	Referent	24 (21.4)	Referent	4 (5.0)	Referent	29 (33.0)	Referent
Light	137 (15.7)	112 (15.2)	1.32 (0.93–1.87)	62 (13.5)	0.86 (0.57–1.30)	19 (17.0)	3.44 (1.61–7.32)	17 (21.5)	11.27 (3.50–36.26)	14 (15.9)	1.47 (0.71–3.04)
Moderate	49 (5.6)	76 (10.3)	1.88 (1.20–2.96)	41 (8.9)	1.41 (0.83–2.39)	15 (13.4)	6.09 (2.62–14.15)	13 (16.5)	18.94 (5.45–65.73)	7 (8.0)	1.09 (0.40–2.98)
Heavy	55 (6.3)	111 (15.1)	1.89 (1.25–2.85)	59 (12.9)	1.09 (0.68–1.75)	21 (18.8)	5.54 (2.47–12.44)	17 (21.5)	16.36 (4.94–54.15)	14 (15.9)	2.64 (1.20–5.81)
Did not drink liquor but drank other types of alcohol	189 (21.7)	205 (27.8)	1.52 (1.12–2.06)	122 (26.6)	1.14 (0.80–1.62)	32 (28.6)	3.78 (1.91–7.48)	28 (35.5)	11.68 (3.85–35.42)	23 (26.1)	1.46 (0.76–2.79)
Unknown	7 (0.8)	11 (1.5)	—	9 (2.0)	—	1 (0.9)	—	0 (0.0)	—	1 (1.1)	—
Every 10 grams/day of ethanolfrom liquor			1.04 (1.01–1.08)		1.03 (0.99–1.07)		1.07 (1.00–1.14)		1.08 (1.00–1.16)		1.07 (1.01–1.14)
Every 10 grams/day of ethanolfrom other types of alcoholic beverage			1.03 (1.00–1.06)		1.01 (0.98–1.04)		1.05 (1.01–1.09)		1.06 (1.02–1.11)		1.04 (0.99–1.08)

Abbreviations: CI = confidence interval; N = number; OR = odds ratio.

^a^Light: <14 grams/day; moderate: 14–42 grams/day; heavy: >42 grams/day.

^b^OR and 95% CI were calculated using unconditional logistic regression, adjusted for sex, age, education, cigarette smoking (pack-year categories) and betel quid chewing (pack-year categories).

The HNC risk was associated with ethanol metabolizing gene polymorphisms (Table 4). For *ADH1B* rs1229984, an increased HNC risk was observed for the *1/*1 genotype (OR = 2.10, 95% CI: 1.37–3.21). For *ALDH2* rs671, an increased HNC risk was observed for the heterozygous *1/*2 genotype (OR = 1.63, 95% CI: 1.28–2.08). In the analysis combining *ADH1B* and *ALDH2*, an increased HNC risk was observed for Group 2 (fast ADH1B*2/*2 + *ALDH2* slow *1/*2 or non-functional *2/*2) and Group 4 (slow ADH1B *1/*2 or *1/*1 + *ALDH 2* slow *1/*2 or non-functional *2/*2) individuals.

**Table 4:** Head and neck cancer risk associated with *ALDH2* rs671, and *ADH1B* rs1229984.

	Cases N = 782 n (%)	Controls N = 915 n (%)	OR (95% CI)^a^
***ADH1B*** **rs1229984**			
TT (*2/*2)(Fast)	405 (51.8)	484 (52.9)	Referent
CT (*1/*2)	290 (37.1)	381 (41.6)	0.91 (0.72–1.15)
CC (*1/*1)(Slow)	87 (11.1)	50 (5.5)	2.10 (1.37–3.21)
***ALDH2*** **rs671**			
GG (*1/*1)(Fast)	325 (41.6)	447 (48.8)	Referent
AG (*1/*2)	403 (51.5)	386 (42.2)	1.63 (1.28–2.08)
AA (*2/*2) (Non-functional)	54 (6.9)	82 (9.0)	1.23 (0.77–1.97)
***ADH1B*** **rs1229984 + ** ***ALDH2*** **rs671**			
Group 1: Fast *ADH1B* (*2/*2) + Fast *ALDH2* (*1/*1)	175 (22.4)	249 (27.2)	Referent
Group 2: Fast *ADH1B* (*2/*2) + Slow *ALDH2* (*1/*2 + *2/*2)	230 (29.4)	235 (25.7)	1.68 (1.22–2.33)
Group 3: Slow *ADH1B* (*1/*1 + *1/*2) + Fast *ALDH2* (*1/*1)	150 (19.2)	198 (21.6)	1.09 (0.79–1.52)
Group 4: Slow *ADH1B* (*1/*1 + *1/*2) + Slow *ALDH2* (*1/*2 + *2/*2)	227 (29.0)	233 (25.5)	1.63 (1.18–2.23)

Abbreviations: CI = confidence interval; N = number; OR = odds ratio.

^a^OR and 95% CI were calculated using unconditional logistic regression, adjusted for sex, age, education, cigarette smoking (pack-year categories), betel quid chewing (pack-year categories), and alcohol drinking (frequency).

The association between alcohol and HNC risk differed by *ADH1B* and *ALDH2* polymorphisms (Table 5). The largest increase in HNC risk associated with alcohol was observed in Group 4 (slow ADH1B*1/*2 or *1/*1 + *ALDH 2* slow *1/*2 or non-functional *2/*2) individuals (OR = 4.26, 95% CI: 2.68–6.78) followed by Group 2 (fast ADH1B*2/*2 + *ALDH2* slow *1/*2 or non-functional *2/*2) individuals (OR = 2.16, 95% CI: 1.34–3.48) (*P*-interaction < 0.0001). For group 4 individuals, even weekly or light drinking may increase HNC risk significantly. Group 4 individuals also showed a positive association between ever regular alcohol drinking and oral cancer, which was not evident in the analysis not stratified by genotypes.

**Table 5:** The association between alcohol drinking and head and neck cancer risk by the combination of *ALDH2* rs671 and *ADH1B* rs1229984.

Ethanol metabolism group								
Alcohol consumption^a^	Group 1 Fast *ADH1B* (*2/*2) + Fast *ALDH2* (*1/*1)		Group 2 Fast *ADH1B* (*2/*2) Slow/non-functional *ALDH2* (*1/*2 + *2/*2)		Group 3 Slow *ADH1B* (*1/*1 + *1/*2) Fast *ALDH2* (*1/*1)		Group 4 Slow *ADH1B* (*1/*1 + *1/*2) Slow/non-functional (*1/*2 + *2/*2)	
	Ca/Co	OR (95% CI)^b^	Ca/Co	OR (95% CI)^b^	Ca/Co	OR (95% CI)^b^	Ca/Co	OR (95% CI)^b^
**All head and neck cancer**								
Never + occasional	44/84	Referent	109/180	Referent	27/68	Referent	71/169	Referent
Ever regular	131/165	0.57 (0.31–1.03)	121/55	2.16 (1.34–3.48)	123/130	1.50 (0.74–3.04)	156/64	4.26 (2.68–6.78)
	*P*-interaction < 0.0001							
Never	37/70	Referent	103/162	Referent	21/51	Referent	65/156	Referent
Monthly	7/14	0.93 (0.31–2.83)	6/18	0.60 (0.20–1.79)	6/17	1.29 (0.36–4.63)	6/13	1.66 (0.56–4.93)
Weekly	24/49	0.49 (0.22–1.08)	21/19	1.51 (0.70–3.28)	19/35	1.67 (0.63–4.42)	18/22	2.43 (1.12–5.26)
Daily	101/116	0.56 (0.29–1.10)	98/33	2.51 (1.45–4.32)	98/93	1.56 (0.66–3.71)	134/41	5.27 (3.15–8.85)
Unknown	6/0	—	2/3	—	6/2	—	4/1	—
	*P*-interaction < 0.0001^c^							
Never	37/68	Referent	101/153	Referent	18/49	Referent	54/150	Referent
Light	29/66	0.52 (0.25–1.09)	27/35	1.16 (0.60–2.24)	20/54	1.04 (0.39–2.77)	26/32	2.50 (1.26–4.97)
Moderate	24/43	0.58 (0.26–1.27)	25/11	1.38 (0.59–3.24)	30/34	1.86 (0.69–5.07)	30/14	4.17 (1.90–9.15)
Heavy	62/54	0.65 (0.30–1.39)	61/16	2.74 (1.37–5.51)	59/39	2.14 (0.79–5.75)	87/20	8.22 (4.31–15.67)
Unknown	7/0	—	2/4	—	6/4	—	3/2	—
	*P*-interaction < 0.0001^c^							
Every 10 grams/day		1.01 (0.98–1.03)		1.05 (0.99–1.11)		1.05 (1.00–1.10)		1.13 (1.07–1.19)
	*P*-interaction = 0.002							
**Oral cavity**								
Never + occasional	29/84	Referent	84/180	Referent	26/68	Referent	52/169	Referent
Ever regular	91/165	0.59 (0.29–1.23)	65/55	1.40 (0.81–2.41)	82/130	0.83 (0.37–1.87)	72/64	2.42 (1.43–4.09)
	*P*-interaction = 0.004							
Never	27/70	Referent	81/162	Referent	20/51	Referent	46/156	Referent
Monthly	2/14	0.38 (0.07–2.05)	3/18	0.34 (0.08–1.41)	6/17	1.24 (0.32–4.84)	6/13	2.32 (0.75–7.07)
Weekly	16/49	0.43 (0.17–1.10)	14/19	1.25 (0.50–3.10)	12/35	0.91 (0.28–2.94)	7/22	1.17 (0.42–3.27)
Daily	70/116	0.50 (0.22–1.13)	49/33	1.41 (0.76–2.62)	64/93	0.81 (0.30–2.20)	63/41	3.11 (1.74–5.54)
Unknown	5/0	—	2/3	—	6/2	—	2/1	—
	*P*-interaction = 0.005^c^							
Never	27/68	Referent	79/153	Referent	17/49	Referent	38/150	Referent
Light	21/66	0.43 (0.18–1.03)	18/35	1.04 (0.48–2.23)	14/54	0.55 (0.17–1.83)	14/32	1.65 (0.72–3.77)
Moderate	17/43	0.52 (0.20–1.33)	12/11	0.78 (0.29–2.08)	20/34	0.85 (0.25–2.90)	17/14	3.25 (1.35–7.82)
Heavy	40/54	0.49 (0.19–1.23)	31/16	1.51 (0.69–3.27)	35/39	0.71 (0.22–2.32)	35/20	4.11 (1.98–8.54)
Unknown	6/0	—	2/4	—	6/4	—	1/2	—
	*P*-interaction = 0.01^c^							
Every 10 grams/day		1.00 (0.97–1.03)		1.01 (0.97–1.06)		1.02 (0.97–1.07)		1.08 (1.03–1.14)
	*P*-interaction = 0.11							
**Oropharynx**								
Never + occasional	5/84	Referent	10/180	Referent	1/68	Referent	8/169	Referent
Ever regular	16/165	1.02 (0.24–4.34)	23/55	6.69 (2.38–18.86)	16/130	18.50 (1.43–240.03)	29/64	9.49 (3.22–27.93)
	*P*-interaction = 0.14							
Never	3/70	Referent	9/162	Referent	1/51	Referent	8/156	Referent
Monthly	2/14	4.76 (0.57–39.96)	1/18	1.35 (0.15–12.61)	0/17	—	0/13	—
Weekly	3/49	1.68 (0.22–12.50)	2/19	2.58 (0.43–15.46)	5/35	16.11 (1.11–233.63)	3/22	2.73 (0.48–15.62)
Daily	12/116	1.76 (0.29–10.77)	21/33	9.51 (3.10–29.22)	11/93	14.54 (0.94–224.84)	25/41	11.96 (3.74–38.31)
Unknown	1/0	—	0/3	—	0/2	—	1/1	—
	*P*-interaction = 0.52^c^							
Never	3/68	Referent	9/153	Referent	1/49	Referent	6/150	Referent
Light	3/66	1.57 (0.21–11.73)	2/35	1.29 (0.24–7.02)	4/54	8.68 (0.60–125.25)	4/32	5.23 (0.95–28.73)
Moderate	4/43	4.49 (0.58–34.60)	5/11	5.80 (1.25–27.04)	5/34	23.89 (1.66–344.63)	3/14	5.66 (0.91–35.09)
Heavy	7/54	3.38 (0.40–28.57)	16/16	15.23 (4.17–55.60)	7/39	21.50 (1.34–346.11)	21/20	32.10 (7.64–134.95)
Unknown	1/0	—	0/4	—	0/4	—	1/2	—
	*P*-interaction = 0.48^c^							
Every 10 grams/day		1.02 (0.95–1.10)		1.12 (1.03–1.22)		1.09 (0.99–1.21)		1.13 (1.05–1.22)
	*P*-interaction = 0.06							
**Hypopharynx**								
Never + occasional	2/84	Referent	2/180	Referent	0/68	Referent	0/169	Referent
Ever regular	10/165	0.35 (0.04–3.07)	21/55	39.04 (7.53–202.44)	12/130	—	41/64	—
	*P*-interaction = ^d^							
Never	2/70	Referent	2/162	Referent	0/51	Referent	0/156	Referent
Monthly	0/14	—	0/18	—	0/17	—	0/13	—
Weekly	3/49	0.70 (0.06–8.48)	3/19	20.30 (2.60–158.68)	0/35	—	6/22	—
Daily	7/116	0.22 (0.02–2.21)	18/33	44.00 (7.93–244.10)	12/93	—	35/41	—
Unknown	0/0	—	0/3	—	0/0	—	0/1	—
	*P*-interaction = ^d^							
Never	2/68	Referent	2/153	Referent	0/49	Referent	0/150	Referent
Light	2/66	0.22 (0.02–3.07)	3/35	5.96 (0.82–43.62)	0/54	—	7/32	—
Moderate	1/43	0.13 (0.01–2.92)	6/11	63.16 (6.58–606.45)	3/34	—	9/14	—
Heavy	6/54	0.62 (0.06–6.52)	7/16	51.30 (5.59–470.69)	9/39	—	21/20	—
Unknown	0/0	—	0/4	—	0/4	—	0/2	—
	*P*-interaction = ^d^							
Every 10 grams/day		1.04 (0.98–1.11)		1.10 (1.00–1.21)		1.13 (1.01–1.26)		1.15 (1.06–1.24)
	*P*-interaction = 0.07							
**Larynx**								
Never + occasional	8/84	Referent	13/180	Referent	0/68	Referent	11/169	Referent
Ever regular	14/165	0.21 (0.06–0.77)	12/55	2.01 (0.73–5.56)	13/130	—	14/64	4.49 (1.58–12.76)
	*P*-interaction = ^d^							
Never	5/70	Referent	11/162	Referent	0/51	Referent	11/156	Referent
Monthly	3/14	3.41 (0.34–34.53)	2/18	2.45 (0.46–13.17)	0/17	—	0/13	—
Weekly	2/49	0.13 (0.02–1.08)	2/19	1.93 (0.35–10.68)	2/35	—	2/22	2.09 (0.36–12.27)
Daily	12/116	0.38 (0.09–1.73)	10/33	2.68 (0.84–8.51)	11/93	—	11/41	5.40 (1.68–17.36)
Unknown	0/0	—	0/3	—	0/2	—	1/1	—
	*P*-interaction = ^d^							
Never	5/68	Referent	11/153	Referent	0/49	Referent	10/150	Referent
Light	3/66	0.22 (0.03–1.45)	4/35	1.95 (0.52–7.26)	2/54	—	1/32	0.80 (0.08–7.56)
Moderate	2/43	0.12 (0.01–1.07)	2/11	1.25 (0.18–8.69)	2/34	—	1/14	1.54 (0.12–20.02)
Heavy	9/54	1.06 (0.20–5.69)	7/16	3.46 (0.89–13.42)	8/39	—	10/20	11.84 (2.88–48.63)
Unknown	0/0	—	0/4	—	0/4	—	1/2	—
	*P*-interaction = ^d^							
Every 10 grams/day		1.03 (0.98–1.08)		1.02 (0.93–1.12)		1.13 (1.03–1.23)		1.13 (1.04–1.22)
	*P*-interaction = 0.16							

Abbreviations: Ca = Case; CI = confidence interval; Co = control; N = number; OR = odds ratio

^a^Light: < 14 grams/day; moderate: 14-42 grams/day; heavy: > 42 grams/day

^b^OR and 95% CI were calculated using unconditional logistic regression, adjusted for sex, age, education, cigarette smoking (pack-year categories), and betel quid chewing (pack-year categories)

^c^Subjects with unknown status of alcohol consumption were excluded for the calculation of *P*-interaction.

^d^ *P*-interaction was not calculated because the ORs in some strata could not be calculated.

Overall, 21.8% of the HNCs, 55.7% of the oropharyngeal cancers, and 89.1% of the hypopharyngeal cancers in our study population could be attributed to ever regular alcohol drinking (Table 6). The population attributable risk percent (PAR%) varied by *ADH1B* and *ALDH2* polymorphisms. Approximately 47.3% of the HNCs among Group 4 (slow ADH1B*1/*2 or *1/*1 + *ALDH 2* slow *1/*2 or non-functional *2/*2) individuals could be attributed to ever regular alcohol drinking. Although oral cancer overall was not significantly associated with alcohol, 28.1% of the oral cancers among Group 4 individuals could be attributed to ever regular alcohol drinking.

**Table 6:** Population attributable risk percent (PAR%) associated with ever drinking alcohol regularly for head and neck cancer overall and by different sites according to the combinations of *ALDH2* rs671 and *ADH1B* rs1229984.

	Overall	Ethanol metabolism group			
		Group 1Fast *ADH1B* (*2/*2) + Fast *ALDH2* (*1/*1)	Group 2Fast *ADH1B* (*2/*2) + Slow/non-functional *ALDH2* (*1/*2 + *2/*2)	Group 3Slow *ADH1B* (*1/*1 + *1/*2) + Fast *ALDH2* (*1/*1)	Group 4Slow *ADH1B* (*1/*1 + *1/*2) + Slow/non-functional *ALDH2* (*1/*2 + *2/*2)
Head and neck cancer	21.8%	^a^	21.3%	^a^	47.3%
Oral cancer	^a^	^a^	^a^	^a^	28.1%
Oropharyngeal cancer	55.7%	^a^	57.1%	92.0%	70.0%
Hypopharyngeal cancer	89.1%	^a^	89.9%	^b^	^b^
Laryngeal cancer	^a^	^a^	^a^	^b^	49.0%

^a^PAR% was not calculated because the association was not statistically significant or the association showed a decreased risk associated with alcohol drinking.

^b^PAR% was not calculated because the OR was not calculable due to zero count in the referent group.

After ≧10 years of cessation from regular alcohol drinking, the HNC risk decreased to the level of never + occasional drinkers (Table 7). The HNC risk reduction was more significant for former light or moderate drinkers than for former heavy drinkers.

**Table 7:** The association between alcohol cessation and risk of head and neck cancer.

Cessation from alcohol drinking	All subjects			Former drinkers were light or moderate drinkers			Former drinkers were heavy drinkers		
	Case n (%)	Control n (%)	OR (95% CI)^a^	Case n (%)	Control n (%)	OR (95% CI)^a^	Case n (%)	Control n (%)	OR (95% CI)^a^
Current regular drinker	437 (53.9)	314 (33.4)	Referent	398 (58.8)	294 (35.1)	Referent	398 (58.2)	294 (36.7)	Referent
< 5 years	50 (6.2)	32 (3.4)	0.76 (0.46–1.26)	16 (2.4)	17 (2.0)	0.57 (0.26–1.24)	26 (3.8)	11 (1.4)	1.08 (0.50–2.33)
5–9.9 years	23 (2.8)	25 (2.7)	0.79 (0.42–1.50)	11 (1.6)	16 (1.9)	0.76 (0.32–1.79)	10 (1.4)	5 (0.6)	1.10 (0.35–3.45)
> 10 years	34 (4.2)	46 (4.9)	0.46 (0.27–0.79)	13 (1.9)	32 (3.8)	0.31 (0.14–0.65)	15 (2.2)	11 (1.4)	0.75 (0.30–1.87)
Never + occasional drinkers	263 (32.4)	517 (55.0)	0.55 (0.43–0.71)	235 (34.7)	476 (56.9)	0.54 (0.41–0.71)	235 (34.4)	476 (59.5)	0.54 (0.42–0.71)
Unknown	4 (0.5)	6 (0.6)	—	4 (0.6)	2 (0.2)	—	0 (0.0)	3 (0.4)	—

Abbreviations: CI = confidence interval; N = number; OR = odds ratio

aOR and 95% CI were calculated using unconditional logistic regression, adjusted for sex, age, education, cigarette smoking (pack-year categories) and betel quid chewing (pack-year categories).

## Discussion

In our analysis, alcohol showed a positive dose-response relationship with HNC risk, with the highest risk observed for hypopharyngeal cancer, followed by oropharyngeal cancer, and laryngeal cancer. With the same amount of pure alcohol, liquor showed a stronger positive association with HNC risk. The HNC risk associated with alcohol was the highest for individuals with the slow *ADH1B* and slow/non-functional *ALDH2* genotype combination followed by individuals with the fast *ADH1B* and slow/non-functional *ALDH2* genotype combination. Overall, alcohol may account for 21.8% of HNCs, 55.7% of the oropharyngeal cancers, and 89.1% of the hypopharyngeal cancers in our study population. Approximately 47.3% of the HNCs among individuals with the slow *ADH1B* and slow/non-functional *ALDH2* genotype combination could be attributed to alcohol drinking. Ten or more years of cessation from regular alcohol drinking was associated with a reduced HNC risk comparable to that of never + occasional drinkers.

We observed a positive association between alcohol and HNC, with the strongest association for hypopharyngeal cancer followed by oropharyngeal cancer. Previous studies also reported that HNC occurring at the pharynx showed the strongest association with alcohol^4^. In Taiwan, among the HNCs occurring at HPV-unrelated sites, the incidence of hypopharyngeal cancer has risen the fastest^29^, and this may be explained by the rising alcohol consumption. The prevalence of cigarette smoking in Taiwan reduced from 31% in 1971 to 20% in 2010^30^. The consumption of betel quid, which is more strongly associated with oral cancer^31, 32^, peaked in 1996 in Taiwan and decreased thereafter^33^. In contrast, alcohol consumption in Taiwan began to increase from 1970’s to late 1990’s/early 2000’s^34^. It is unclear why hypopharynx is more susceptible to the carcinogenic effect of alcohol compared to other head and neck sites and more investigations are needed.

Current literature is inconsistent on the association between HNC risk and different alcoholic beverage types. In our analysis, liquor showed a stronger positive association with HNC risk compared to beer and wine, although the result for wine needs to be examined with caution due to the small percentage of wine drinkers in our study population. In agreement with our study, three published studies also reported a higher HNC risk associated with liquor than with beer or wine^5, 9, 10^. In contrast, two studies showed that HNC risk did not vary significantly by alcoholic beverage type^8, 12^. Other studies reported that HNC risk varied by alcoholic beverage type, but the type most strongly associated with HNC was different^6, 7, 11, 13^. It was suggested that the alcoholic beverage type most commonly consumed would be the one most strongly associated with HNC^11^. However, in our study, beer was more commonly consumed than liquor but its association with HNC risk was weaker than that between liquor and HNC. In addition to the acetaldehyde produced by ethanol metabolisms, many alcoholic beverages already contain acetaldehyde, which is higher in spirits and fortified wine^35^. However, after standardizing to alcohol strength, the acetaldehyde contents in beer, wine, and spirits are not significantly different^35^. Therefore, acetaldehyde contents in alcoholic beverages do not explain our finding. Given the inconsistent findings across studies, it is inconclusive whether the HNC risk varies by alcoholic beverage type.

The polymorphism of *ALDH2* has been shown to affect drinking behavior while the polymorphism of *ADH1B* showed less influence^36^. The carriers of ALDH2*2 allele tend to drink less due to the unpleasant symptoms associated with slow acetaldehyde clearance, including facial flushing, palpitation, tachycardia, and nausea^37^. However, even though carriers of ALDH2*2 allele may drink less, they are more susceptible to the carcinogenic effect of alcohol than carriers of ALDH2*1/*1. *ALDH2* along with *ADH1B* may modify the association between alcohol and HNC risk as seen in our analysis. After alcohol consumption, most ethanol is absorbed by the intestines and some is metabolized by liver, while some is recirculated through the bloodstream to the salivary glands^38^. Some of the ethanol is converted to acetaldehyde in the salivary glands through human ethanol metabolism, while some ethanol is released with saliva into the oral cavity, where oral microorganisms can covert ethanol to acetaldehyde^38, 39^. In our analysis, the HNC risk was not increased by alcohol for group 1 individuals (fast *ADH1B + *fast *ALDH2*), likely due to efficient ethanol metabolism. Alcohol was associated with a significantly increased HNC risk for group 2 individuals (fast *ADH1B* + slow/non-functional *ALDH2*), likely due to the rapid production and accumulation of acetaldehyde. The highest HNC risk associated with alcohol was observed in Group 4 individuals (slow *ADH1B* + slow/non-functional *ALDH2*). Besides acetaldehyde production by human ethanol metabolism, Group 4 individuals are also more likely to be exposed to the acetaldehyde produced by oral microorganisms. The ethanol and acetaldehyde levels were found to be higher in individuals with slow *ADH1B* genotype compared to individuals with fast *ADH1B* genotype^40^. While the ethanol levels were similar in the saliva and blood, the acetaldehyde level in the saliva was much higher than in the blood, suggesting additional acetaldehyde production by oral microorganisms^40^. This was supported by the correlation between salivary acetaldehyde levels and the oral microorganism counts^40^. The oral microorganism counts and salivary acetaldehyde all decreased after three weeks of alcohol abstinence^40^.

In our study population, which consisted of mostly men (~95% of HNCs), we estimated that approximately 22% of HNCs could be attributed to alcohol consumption. PAR% is influenced by the prevalence of exposure and the strength of the association between exposure and disease^41^. In a previous study on the alcohol attributable burden of cancer using data from eight European countries, it was estimated that 37% to 47% of the upper aerodigestive tract (UADT) cancer (HNC + esophageal cancer) among European men could be attributed to alcohol drinking^42^. The percentages of never drinkers among the European men in this study were all quite low at less than 10% and consequently the percentages of UADT cancer attributable to alcohol drinking were high. Among the individuals with slow ethanol metabolism (slow *ADH1B* + slow/non-functional *ALDH2*) in our study, because the strength of association between alcohol and HNC was stronger, the percent of HNC attributable to alcohol was also high at 47%. Although compared to the Western populations, the PAR% of HNC attributed to alcohol is overall lower in our study population due to the higher percentage of never drinkers, because of the heightened susceptibility of individuals with slow ethanol metabolism to the carcinogenic effect of alcohol, alcohol may contribute to a higher percentage of HNCs in these individuals.

We found that the HNC risk of former regular alcohol drinkers became comparable to that of never + occasional drinkers after ≧10 years of cessation from regular alcohol drinking. A meta-analysis reported that every year of alcohol cessation was associated with a 2% reduction in the risk of pharyngeal and laryngeal cancers^17^. However, in contrast to our result, this meta-analysis showed that it took 36 years and 39 years of alcohol cessation, respectively, for the risk of laryngeal cancer and pharyngeal cancer to reduce to the level of never drinkers^17^. One possibility for these discrepancies may be the level of alcohol consumption before alcohol cessation. We found that the reduced HNC risk associated with alcohol cessation was more significant for former light or moderate drinkers than for former heavy drinkers. Different levels of alcohol consumption in different regions of the world may explain the discrepancies regarding the effect of alcohol cessation on HNC risk. Heavy drinking may cause more tissue damage and thus need longer period of alcohol cessation to repair the damage. Currently, there is a paucity of data in the literature regarding the impact of alcohol consumption level before alcohol cessation on the association between alcohol cessation and HNC risk and more studies are required.

This study has several limitations. It is often difficult to determine in a hospital-based case-control study whether cases and controls are from the same source population but several steps were taken to minimize selection bias. First, our recruiting hospital is a major medical center in Tainan City and 97% of the study subjects came from Tainan City and the neighboring areas. Second, we excluded from controls those with diseases that might be related to the use of alcohol, betel quid, and cigarette. Third, the percentage of ever regular alcohol drinker among our controls (45%) is similar to a population survey that reported a percentage of ever alcohol drinker ranging from 42% to 49% in Tainan City^43^. However, information was not available to compare the amount of drinking. Despite these efforts, this hospital-based case-control study is still prone to selection bias. Since our recruiting hospital is a major referral medical center in Southern Taiwan, our HNC cases might not represent the HNC cases occurring among the general population in the area. In addition, our controls were patients who required surgery for non-cancerous diseases of the head and neck, with 41.7% with benign lesions of the head and neck. These hospital-based controls were likely to have health behaviors different from those of the population-based controls. For example, it has been shown that hospital-based controls tend to include more heavy drinkers than population-based controls^44^. In such case, our results could have been biased towards the null and underestimated the true positive association between alcohol consumption and HNC. Recall bias is another concern. The HNC subjects might have ruminated more about their alcohol drinking and over-reported alcohol consumption, biasing the results away from the null. Information bias is another limitation because the internal validity of the questions for determining the level (grams of alcohol per day) of alcohol consumption was not validated. The grams of alcohol were estimated indirectly according to the volume and the types of alcohol consumed. Consequently, the risk estimates associated with the level of alcohol consumption might lack precision and could only reflect a general trend for the association between alcohol consumption level and HNC risk. In the analyses stratified by HNC sites, genotypes and types of alcoholic beverage, the sample sizes in some of the groups were small and resulted in imprecise estimates. Finally, for the analysis with smoking cessation, it is possible that other co-morbidities may be the motivation to quit smoking and may be a confounder in the association between smoking cessation and HNC risk. Unfortunately, we could not adjust for other co-morbidities in our analysis because we did not collect such information.

This study has several strengths. First, we analyzed oropharyngeal and hypopharyngeal cancers separately and found that among all HNCs, hypopharyngeal cancer showed the strongest association with alcohol. This allowed us to correlate the increasing alcohol consumption with the rapid rise of hypopharyngeal cancer incidence in Taiwan. Second we combined the polymorphisms of *ADH1B* and *ALDH2* for a complete evaluation of gene-alcohol interaction on HNC risk.

Overall, we observed a positive dose-response association between alcohol and HNC and the association was the strongest for hypopharyngeal cancer, followed by oropharyngeal cancer, and laryngeal cancer. Liquor had a larger impact in HNC risk than wine and beer, although this needs further confirmation. *ADH1B* and *ALDH2* polymorphisms may modify the HNC risk associated with alcohol, and the highest risk was among individuals with the slow *ADH1B* and slow/non-functional *ALDH2* genotype combination followed by individuals with the fast *ADH1B* and slow/non-functional *ALDH2* genotype combination. The HNC risk became comparable to that of never + occasional drinkers after ≧10 years of cessation from regular alcohol drinking. For the purpose of reducing HNC incidence, public health program should be implemented to promote reduction or cessation of alcohol drinking. However, low to moderate alcohol consumption may be beneficial to prevent cardiovascular diseases^45^. More investigations are needed to determine the minimal acceptable dose of alcohol, taking into consideration both the harmful and beneficial effect of alcohol. Particular attention should be paid to individuals with slow ethanol metabolism because these individuals may have an increased susceptibility to the carcinogenic effect of alcohol.

## Methods

The institutional review boards of the National Cheng Kung University Hospital and the National Health Research Institutes approved the current study. A signed informed consent was obtained from every study participant. Recruitment and interview of human subjects were performed in accordance with guidelines approved by the National Cheng Kung University Hospital and the National Health Research Institutes.

### Study subjects

For this study, we used a hospital-based case-control study design. Cases were primary HNC patients treated at the National Cheng Kung University Hospital from September 1, 2010 to August 29, 2016. The eligibility criteria for cases included: 1) pathologically confirmed squamous cell carcinoma of head and neck (oral cavity, oropharynx, hypopharynx, and larynx) (ICD-10 codes: C00-C10, C12-C14, C32); 2) no previous cancer diagnosis; 3) age ≧ 20 years; and 4) ability to provide informed consent. For comparison, controls frequency-matched to the cases by age (±5 years) and sex were recruited from the department of otolaryngology and the department of stomatology. The eligibility criteria for controls were: 1) diagnosis of non-cancerous head and neck diseases that required surgery and were unrelated to the use of alcohol, betel quid, and cigarette; 2) no previous cancer diagnosis; 3) age ≧ 20 years; and 4) ability to provide informed consent.

### Data collection

Alcohol consumption data were obtained from study subjects by trained interviewers using a standardized questionnaire, which was pilot tested for the subjects’ understanding of the questions, the capability of the questions to capture the subjects’ exposure histories, and for the interpretability of the responses. Each study subject was first asked whether he/she ever drank alcohol. Those with a positive response were further asked the followings: (1) starting age; (2) quitting age if the subject had quit drinking alcohol for >6 months; (3) type of alcoholic beverage (beer, wine, and liquor); and (4) average drinking frequency (monthly, weekly, or daily) in their lifetime before the diagnosis of HNC or before the interview date (for control subjects) and the number of cups (1 cup = 150 ml) consumed each time. The volume 150 ml is the size of the paper cup we used to show the study subjects as the reference to help them recall the volume of alcohol consumed. Frequency was determined by average. For example, if someone drank four times per month, this would average out to once per week and be categorized as weekly drinking. Data on potential confounders, including sex, age, education, use of betel quid and cigarette, and consumption of vegetables and fruits were also collected. Sex and age are considered universal confounders on the association between various exposures and diseases. Education as a marker of socioeconomic status is often a confounder on the association between lifestyle factors and diseases. In Taiwan, alcohol drinkers often also use cigarette and betel quid, both of which are independent risk factors of HNC. Finally, alcohol drinkers may live an unhealthier lifestyle and consume less vegetables and fruits. Higher intake of vegetables and fruits has been independently associated with a reduced HNC risk^46^.

### Blood sample collection

Pre-treatment blood sample was collected from each study subject in an EDTA-containing vacutainer tube. The blood sample was centrifuged to obtain the buffy coat, from which the genomic DNA was extracted using commercially available DNA purification kit. DNA samples were stored in a −80 °C refrigerator until ready to use.

### Genotyping

*ADH1B* rs1229984 and *ALDH2* rs671 were genotyped by Taqman-based allelic discrimination method on an Applied Biosystems 7500 Real-Time Polymerase Chain Reaction System (Applied Biosystems, Foster City, CA). For quality control, 10% of the samples were randomly selected for duplicate genotyping and the concordance was 100%.

### Statistical analysis

The distributions of sex, age, education, use of alcohol and betel quid, and consumption of vegetables and fruits were compared between cases and controls using T-tests (for continuous variables) and chi-squared tests (for categorical variables). Unconditional logistic regression was performed to estimate the odds ratio (OR) and 95% confidence interval (CI) for the association between alcohol and HNC, adjusted for sex, age, education, and use of cigarette and betel quid. Even though case and controls were frequency matched on sex and age, we adjusted for sex and age to account for the residual imbalance of these variables between cases and controls after the frequency-matching process. We initially adjusted for the consumption of fruits and vegetables, but it changed the OR estimates by <5% (data not shown) and we decided to exclude it from the final statistical models.

Alcohol consumption was evaluated in several ways: (1) by status: never + occasional drinker, former regular drinker, and current regular drinker. Regular drinking = drinking alcohol for at least once per week; (2) by frequency: never, monthly, weekly, and daily; (3) by the amount: the amount of alcohol consumed per day in grams was calculated according to alcoholic beverage type, volume, and frequency. According to the drinking frequency, the number of cups per day was determined for each beverage type. The volume per day of each beverage type was determined by the total number of cups (1 cup = 150 ml) per day consumed for the beverage type. The grams/day for each beverage type was calculated with the formula: total volume per day x alcohol content × 0.798 g/ml. 0.798 g/ml is the density of ethanol. The alcohol content was set at 5% for beer, 13% for wine, and 40% for liquor. The total grams/day of alcohol was calculated by summing the grams/day of all beverage types. Individuals were then divided into never, light drinker (<14 grams/day), moderate drinker (14–42 grams/day), and heavy drinker (>42 grams/day). In addition to analyzing alcohol amount as a categorical variable, it was also analyzed as a continuous variable for the risk of HNC associated with every 10 grams of alcohol/day; and 4) by alcoholic beverage type (beer, wine, and liquor). All analyses were first performed with all HNC cases and then by different HNC sites.

To evaluate the effect of alcohol cessation on HNC risk, we performed unconditional logistic regression with current drinker as the referent group, adjusted for sex, age, education, and use of cigarette and betel quid. The comparison groups included never + occasional drinkers and those who quit regular drinking for <5 years, 5–9.9 years, 10–14.9 years, and >15 years.

To evaluate the influence of alcohol metabolizing genes on HNC risk associated with alcohol consumption, study subjects were divided into four groups by the genotypes of *ADH1B* rs1229984 and *ALDH2* rs671: group 1: fast *ADH1B* (*2/*2) + fast *ALDH2* (*1/*1); group 2: fast *ADH1B* (*2/*2) + slow (*1/*2) or non-functional (*2/*2) *ALDH2*; 3) group 3: slow *ADH1B* (*1/*2 or *1/*1) + fast *ALDH2* (*1/*1) and; group 4: slow *ADH1B* (*1/*2 or *1/*1) + slow (*1/*2) or non-functional (*2/*2) *ALDH2*. The association between alcohol and HNC was evaluated separately for these four groups. We created these genotype combination groups according to the function of the two SNPs. To evaluate the interaction between alcohol metabolizing genes and alcohol consumption on HNC risk, the heterogeneity between genotype combination groups was assessed by the log-likelihood ratio test comparing the unconditional logistic regression model with the product term (alcohol consumption X genotype combination groups) to the model without the product term.

PAR% was calculated to quantify HNC risk attributed to alcohol drinking using the formula^41^:

$${\rm{PAR}} \% =[({{\rm{P}}}_{{\rm{e}}}(\text{RR}-1))/({{\rm{P}}}_{{\rm{e}}}(\text{RR}-1)+1)]\times 100.$$

Relative risk (RR) was estimated using the OR for the association between alcohol consumption and HNC risk. The prevalence of alcohol drinking (P_e_) was estimated by the percentage of ever regular drinkers among controls.

All analyses were performed using SAS 9.4 (SAS Institute, Inc., Cary, North Carolina USA).

### Data availability statement

The datasets generated during and/or analysed during the current study are available from the corresponding author on reasonable request.

## Electronic supplementary material

Supplementary Table 1
