# Supplementary material for: Investigating the Association between Alcohol and Risk of Head and Neck Cancer in Taiwan
Source: Sci Rep. 2017 Aug 29;7:9701. doi: 10.1038/s41598-017-08802-4 (PMC5574999; doi:10.1038/s41598-017-08802-4)
Supplement: Supplementary file 1 — Supplementary Table 1 [file 41598_2017_8802_MOESM1_ESM.doc]

**Investigating the Association between Alcohol and Risk of Head and Neck Cancer in Taiwan**

Cheng-Chih Huang^1^,*, Jenn-Ren Hsiao^1^,*, Wei-Ting Lee^1^, Yao-Chou Lee^2^, Chun-Yen Ou^1^, Chan-Chi Chang^1^, Yu-Cheng Lu^1^, Jehn-Shyun Huang^3^, Tung-Yiu Wong^3^, Ken-Chung Chen^3^, Sen-Tien Tsai^1^, Sheen-Yie Fang^1^, Jiunn-Liang Wu^1^, Yuan-Hua Wu^4^, Wei-Ting Hsueh^4^, Chia-Jui Yen^5^, Shang-Yin Wu^5^, Jang-Yang Chang^5, 6^, Chen-Lin Lin^7^, Yi-Hui Wang^6^, Ya-Ling Weng^6^, Han-Chien Yang^6^, Yu-Shan Chen^1^, Jeffrey S. Chang^6^

1. Department of Otolaryngology, National Cheng Kung University Hospital, College of Medicine, National Cheng Kung University, 138 Sheng Li Road, Tainan 70456, Taiwan.

2. Division of Plastic and Reconstructive Surgery, Department of Surgery, National Cheng Kung University Hospital, College of Medicine, National Cheng Kung University, 138 Sheng Li Road, Tainan 70456, Taiwan.

3. Department of Stomatology, National Cheng Kung University Hospital, College of Medicine, National Cheng Kung University, 138 Sheng Li Road, Tainan 70456, Taiwan.

4. Department of Radiation Oncology, National Cheng Kung University Hospital, College of Medicine, National Cheng Kung University, 138 Sheng Li Road, Tainan 70456, Taiwan.

5. Division of Hematology/Oncology, Department of Internal Medicine, National Cheng Kung University Hospital, College of Medicine, National Cheng Kung University, 138 Sheng Li Road, Tainan 70456, Taiwan.

6. National Institute of Cancer Research, National Health Research Institutes, 1F No 367, Sheng-Li Road, Tainan 70456, Taiwan.

7. Department of Nursing, National Cheng Kung University Hospital, College of Medicine, National Cheng Kung University, 138 Sheng Li Road, Tainan 70456, Taiwan.

*These authors contributed equally to this work

Correspondence to: Jeffrey S. Chang, MD, PhD, MPH

1F No 367, Sheng-Li Road, Tainan 70456, Taiwan

E-mail: jeffreychang@nhri.org.tw

Tel: 886-6-208-3422 ext 65160; Fax: 886-6-208-3427

Supplementary Table 1. The distribution of clinical diagnoses among controls

| **Clinical diagnosis** | **N = 940**  **n** |
| --- | --- |
| Antrochoanal polyp | 3 |
| Benign ethmoid tumor | 3 |
| Benign hypopharyngeal lesions | 11 |
| Benign laryngeal lesions | 44 |
| Benign maxillary sinus lesions | 7 |
| Benign nasopharyngeal tumor | 4 |
| Benign neck tumor | 10 |
| Benign oral lesions | 40 |
| Benign oropharyngeal lesions | 21 |
| Benign parapharyngeal space tumor | 1 |
| Benign salivary gland tumor | 127 |
| Benign vocal cord lesions | 124 |
| Chest abscess | 1 |
| Cholesteatoma | 12 |
| Chronic corditis | 1 |
| Chronic otitis media | 68 |
| Chronic rhinitis | 23 |
| Chronic sinusitis | 207 |
| Deep neck infection | 4 |
| External auditory canal stenosis | 1 |
| Epiglottic cyst | 16 |
| Epistaxis | 1 |
| Esophageal stenosis | 1 |
| Ethmoid mucocele | 3 |
| Incomplete glottis closure | 2 |
| Laryngocele | 1 |
| Mastoiditis | 2 |
| Middle turbinate headache syndrome | 2 |
| Nasal polyp | 1 |
| Nasal septum deviation | 1 |
| Nasal synechiae | 1 |
| Neck lipoma | 36 |
| Neck lymphangioma | 1 |
| Obstructive sleep apnea | 23 |
| Oroantral fistula | 1 |
| Osteoma | 1 |
| Preauricular sinus | 1 |
| Sialolithiasis | 20 |
| Thyroglossal duct cyst | 15 |
| Tonsillitis | 10 |
| Torus palatinus | 1 |
| Tracheal granuloma | 1 |
| Vocal cord palsy | 8 |
| Vocal cord polyp | 78 |
| Wegener’s granulomatosis | 1 |
